# Phylogenetic relationships among *Staphylococcus* species and refinement of cluster groups based on multilocus data

**DOI:** 10.1186/1471-2148-12-171

**Published:** 2012-09-06

**Authors:** Ryan P Lamers, Gowrishankar Muthukrishnan, Todd A Castoe, Sergio Tafur, Alexander M Cole, Christopher L Parkinson

**Affiliations:** 1 Burnett School of Biomedical Sciences, University of Central Florida College of Medicine, 4000 Central Florida Boulevard, Orlando, FL, 32816, USA; 2 Department of Biochemistry and Molecular Genetics, University of Colorado School of Medicine, 12801 17th Avenue, Aurora, CO, 80045, USA; 3 Stokes Advanced Research Computing Center, Institute for Simulation and Training, University of Central Florida, 3100 Technology Parkway, Orlando, FL, 32826, USA; 4 Department of Biology, University of Central Florida, 4000 Central Florida Boulevard, Orlando, FL, 32816, USA; 5 Current affiliation: Department of Biochemistry and Biomedical Sciences, Michael G. DeGroote Institute for Infectious Disease Research, McMaster University, 1280 Main Street West, Hamilton, ON, L8S 4K1, Canada

## Abstract

**Background:**

Estimates of relationships among *Staphylococcus* species have been hampered by poor and inconsistent resolution of phylogenies based largely on single gene analyses incorporating only a limited taxon sample. As such, the evolutionary relationships and hierarchical classification schemes among species have not been confidently established. Here, we address these points through analyses of DNA sequence data from multiple loci (16S rRNA gene, *dnaJ, rpoB,* and *tuf* gene fragments) using multiple Bayesian and maximum likelihood phylogenetic approaches that incorporate nearly all recognized *Staphylococcus* taxa.

**Results:**

We estimated the phylogeny of fifty-seven *Staphylococcus* taxa using partitioned-model Bayesian and maximum likelihood analysis, as well as Bayesian gene-tree species-tree methods. Regardless of methodology, we found broad agreement among methods that the current cluster groups require revision, although there was some disagreement among methods in resolution of higher order relationships. Based on our phylogenetic estimates, we propose a refined classification for *Staphylococcus* with species being classified into 15 cluster groups (based on molecular data) that adhere to six species groups (based on phenotypic properties).

**Conclusions:**

Our findings are in general agreement with gene tree-based reports of the staphylococcal phylogeny, although we identify multiple previously unreported relationships among species. Our results support the general importance of such multilocus assessments as a standard in microbial studies to more robustly infer relationships among recognized and newly discovered lineages.

## Background

The genus *Staphylococcus* currently contains more than 60 species and subspecies. Many are of clinical, agricultural, and economic interest because they lead to high levels of infection among human populations or agricultural loss within the dairy, swine, and poultry industries. Moreover, multiple species within this genus are common pathogens in non-human animals and thus should be monitored with concern as these animals provide reservoirs for pathogenic bacteria [1-3]. Although seemingly uncommon, host switching is an important mechanism in the evolution of *Staphylococcus*. For example, in *S. aureus,* human-to-poultry [4] and bovine-to-human [3] host switches have been observed. As such, a thorough understanding of species relatedness is a necessity for understanding host-pathogen interactions within this genus [5-7].

Many previous estimates of the staphylococcal phylogeny have been based on single locus gene trees, which in many cases exhibit differing topologies. As such, robust species tree estimations have proved to be difficult. Historically, staphylococcal species identification has been a laborious task, requiring multiple biochemical and genotypic methodologies [6,8]. Fortunately, more efficient and reliable assays based on PCR and DNA sequencing have become commonplace as part of the identification process of novel species (and differentiating closely related species). As with most bacterial systems, the 16S rRNA gene continues to be the most common method for staphylococcal species identification, although its utility is limited due to high sequence similarity among different staphylococcal species [9,10]. For this reason, increased emphasis has recently been devoted towards identifying additional genes for use in species identification that offer greater taxonomic resolution between closely related species, while also limiting the incidence of misidentification. Such genes as *rpoB* (β-subunit of RNA polymerase), *tuf* (elongation factor Tu), and *dnaJ* (heat shock protein 40), have been found useful for the identification of staphylococcal species. With the exception of one study where *dnaJ* and *rpoB* were concatenated and assessed under a single evolutionary model [11], each has only been analyzed singularly in a phylogenetic context.

We targeted two related primary goals in this study. First, we aimed to utilize a multilocus phylogenetic dataset to critically evaluate the proposed cluster groupings of species of *Staphylococcus*, and to amend these groupings to reflect estimates of phylogeny. Second, on a broader scale, we aimed to infer the deeper phylogenetic relationships among cluster groups of all *Staphylococcus* species using multilocus data analyzed under different strategies including concatenated and species-tree methods. We analyzed a large multilocus *Staphylococcus* dataset in multiple ways to thoroughly explore the phylogenetic signal in the data, and provide robust confirmatory evidence for the relationships among species. We first analyzed the combined four-gene dataset using partitioned Bayesian and maximum likelihood analyses, in which a single species tree was inferred. Such probabilistic methods of phylogeny are particularly powerful as they incorporate alternative models of character evolution into the analysis and search for a tree that ultimately maximizes the probability of data given the tree [12,13]. Their accuracy, however, can be dependent on the complexity and biological realism of the models of sequence evolution used.

There is a tradeoff between having enough parameters to accurately capture the complexity of sequence evolution in a multilocus dataset, while not having more parameters than can be accurately estimated from the data [14-17]. We therefore tested multiple differently partitioned model schemes to identify which best fit the multilocus dataset. Generally, we expect such partitioned model analysis of the combined (concatenated) dataset will have the best power for inferring the phylogeny of *Staphylococcus*, as long as basic assumptions of the approach are met. The most important of these assumptions is that all the underlying gene trees are the same as the species tree. There are, however, situations where gene trees and species tree are not the same [18,19], or where systematic error in gene-tree estimation may lead to overconfidence in an incorrect species tree [20]. There is some indication, however, that in such cases, maximum likelihood bootstrap support values may be more sensitive to conflicting phylogenetic signals in the data than Bayesian posterior probability support for nodes, although both concatenated data analysis approaches are likely to experience some error [21-23].

Therefore, we also used an alternative approach to estimate relationships among species of *Staphylococcus* in which gene trees are estimated separately, and jointly considered to estimate an underlying species tree. This approach, called Bayesian Estimation of Species Trees analysis [24], thereby avoids concatenation of multiple loci, and estimates a species tree based on a model that accounts for deep coalescence of gene trees. Although this approach does not specifically model all possible scenarios that may violate the assumptions of the concatenated analysis, comparisons of results between this approach and concatenated analyses provides added perspective on the relative robustness of species-level phylogenetic inferences.

## Methods

### DNA sequence acquisition and alignment

DNA sequences for a total of four genes from 57 staphylococcal species, and two outgroup species (*Macrococcus caseolyticus* - strain JCSJ5402, and *Bacillus subtilis* - strain 168) were downloaded from NCBI's GenBank. For each species included in the analysis, sequences were specifically downloaded from the type strain. The four loci collected included the non-coding 16S rRNA gene, and the three protein coding genes: *dnaJ*, *rpoB,* and *tuf*. The list of all species analyzed in this study with the accession numbers for each of the four gene fragments is given in Additional file 1: Table S1.

Nucleotide sequences were aligned using ClustalW in MEGA 4.1 [25], with manual adjustment to ensure that complete codons remained in tact for downstream analyses. Regions of high variability were omitted from the alignments because assessment of homology was questionable [15]. This was only observed to be the case for *dnaJ* in which nucleotide positions 63–93 in the original sequence was omitted. Additional manual codon adjustment of this region did not improve the alignment and thus, was omitted. Secondary structure predictions (i.e. stem and loop regions) for 16S rRNA gene fragments were estimated using the RNAalifold approach [26,27]. The data matrix and trees have been deposited in TreeBase ([28]; http://purl.org/phylo/treebase/phylows/study/TB2:S12505). Analyses of incongruence length differences (ILD; [29]) among partitions of the dataset were performed using PAUP* 4.0 [30]. Nucleotide diversities and species divergence calculations were performed using MEGA 4.1 [25] and DnaSP v5 [31].

### Nucleotide model selection

Models of nucleotide evolution for each gene and nominal partition of the data were estimated using jModelTest v0.1.1 [32,33] based on Akaike Information Criterion (AIC). For the purpose of model testing (and later partitioned Bayesian analyses) we divided the dataset by gene, and into biologically relevant subsets: coding versus non-coding gene fragments, codon position, and stem versus loop secondary structures (for the 16S rRNA gene fragment). These individual partitions, and the best-fit evolutionary model selected for each partition, are shown in Additional file 2: Table S2.

For analyses of the combined data with partitioned models, we formulated nine different partitioning schemes. These were designed to provide a hierarchical spectrum of model complexity, and parameter richness, with increasing partitioning of biologically reasonable sets of the data (Table 1). The simplest model (MB1) was a single evolutionary model (GTR + ΓI) fit to the entire dataset followed by additional models (MB2-MB9) that were created by the addition of dataset partitions among and within non-coding and coding gene fragments (Table 1).

**Table 1:** Description of alternative model partitioning strategies tested for fit to the combined nucleotide data

**Model name**	**# of partitions**	**# of free model parameters**	**Description of model partitions**
MB1	1	10	Single model for concatenated dataset
MB2	2	13	16S; All protein coding gene fragments (*dnaJ; rpoB; tuf*)
MB3	4	29	Independent partition for each gene fragment (*16S; dnaJ; rpoB; tuf*)
MB4	7	48	16S; two partitions for each gene fragment (codon positions 1 and 2; codon position 3)
MB5	8	62	16S, stems; 16S, loops; two partitions for each gene fragment (codon positions 1 and 2; codon position 3)
MB6	10	78	16S; three partitions for each gene fragment (codon positions 1, 2, and 3, separately)
MB7	11	92	16S, stems; 16S, loops; three partitions for each gene fragment (codon positions 1, 2 and 3, separately)
MB8	3	26	16S, stems; 16S, loops; All protein coding gene fragments (*dnaJ; rpoB; tuf*)
MB9	5	43	16S, stems; 16S, loops; Independent partition for each protein coding gene fragment (*dnaJ; rpoB; tuf*)

### Bayesian phylogenetic analysis

Bayesian inference (BI) was carried out using the Metropolis-Hastings coupled Markov chain Monte Carlo method in MrBayes v3.1.2 [34,35] and BEST v2.3.1 [36]. All Bayesian phylogenetic analyses performed in this study were carried out using the STOKES IBM High Performance Computing Cluster at the University of Central Florida. MPI-enabled versions of MrBayes v3.1.2 and BEST v2.3.1 were compiled and run in parallel [37]. For each BI run, gaps in alignments were treated as missing data. For each MrBayes analysis, two independent BI runs were carried out using random starting trees with one cold chain and three heated chains (following program defaults). Each model was assessed in triplicate with summary statistics being estimated from all runs.

In addition to performing BI runs in MrBayes on the unpartitioned multilocus dataset (using the evolutionary model specified by AIC), eight additional models were assessed where independent models of evolution were applied to different nucleotide regions within the combined dataset (refer to nucleotide model selection section). This was achieved by using the “unlink” command in MrBayes v3.1.2. Each BI run consisted of 4 million generations with every 100 steps being sampled. As verified using Tracer v1.5 [38], stationarity was reached in all BI runs prior to 500 000 generations and a conservative burn-in of 1 million (25%) generations was performed. To verify that additional sampling (i.e., increasing the number of generations) for MrBayes runs would not affect the outcome of the data, a final run of 20 million generations with sampling every 1 000 steps and a burn-in of 4 million generations was performed.

In addition to reconstructing phylogenies using MrBayes v3.1.2, Bayesian phylogenetic reconstruction was also performed using BEST v2.3.1, which is a modified version of MrBayes. BEST was implemented by setting the prior for BEST = 1, and unlinking topologies, branch lengths, and mutation rates across loci. For each independent BEST analysis, four simultaneous runs consisting of 16 chains each were performed for 20 million generations with sampling every 1 000 generations. A prior for theta was set at 0.04, based on the mean estimates of theta for the dataset calculated in DnaSP and MEGA 4.1. Consistent with previous reports [39,40], run convergence was only achieved by setting a uniform prior for branch lengths (prset brlenspr = clock:uniform). As with all BI runs, nucleotide regions were assigned nucleotide substitution models based on AIC, estimated in jModelTest.

### Assessment of BI runs

All partitioning strategies employed using MrBayes were run in triplicate to verify reproducibility while BEST analyses were run two separate times. Subsequently, MrBayes and BEST runs, under each model, were assessed using multiple criteria to determine the success of each model and the overall best-fit model. Bayes factors (BF; 2ΔlnB_10_) were calculated from estimates of the harmonic mean of the posterior distribution of cold chain likelihoods. Consistent with previous reports [14,16,41], we set a cutoff of BF > 10 to support one model over another. Recently, multiple studies have suggested that biases introduced by using the harmonic mean estimator may practically affect model selection using BF [42-45]. Based on our results however, we discuss why such biases are practically tolerable in this study (i.e., model choice has little effect on topology and nodal support).

Akaike weights (Aw) [46] were also used to identify best-fit partitioned models [17]. Initially AIC values were calculated by the equation AIC = −2lnL + 2 k where k equals the total number of free parameters within the model. For small samples sets, where the sample size (n) to free parameter (k) ratio is <40, it has been suggested that a small-sample bias adjustment be applied to the AIC calculation, thus calculating AIC_c_ instead [47,48]. The sample size of the staphylococcal dataset (with outgroups) is 59 and the minimum number of free parameters was 10 for model MB1. As such, the n/k ratio was always <40, so we calculated the AIC_c_ instead. The equation for ${\text{AIC}}_{\text{c}}=-2\text{lnL}+2\text{k}2\text{k}\left(\text{k}\;+\;1\right)/\text{n-k}-1$. The ΔAIC_c_ was then calculated by subtracting the model with the minimum AIC_c_ (AIC_cmin_) (i.e. highest lnL) from the ith model using the equation $\Delta {\text{AIC}}_{\text{ci}}={\text{AIC}}_{\text{ci}}-{\text{AIC}}_{\text{cmin}}$. Following calculations of the ΔAIC_c_ for each model, Aw were calculated using the equation ${\text{Aw=e}}^{\left(-\Delta \text{AICci}/2\right)}/\sum {\text{e}}^{\left(-\Delta \text{AICci}/2\right)}$. By this equation, the relative likelihood of a model given the data is normalized over all models and thus, the greater the Aw for a given model, the greater the relative support for that model [14].

Further assessment of model performance was based on examining the output of model parameters and carried out by analyses of multiple additional features. Posterior distributions of parameters and analysis of trace plots were assessed for failed convergence and stationarity using Tracer v1.5 [38]. Also, because model overparameterization has been linked to estimates of tree length in partitioned Bayesian analyses [49], we also compared tree length estimates among runs.

### Maximum likelihood analysis

Phylogenetic reconstruction using maximum likelihood (ML) analysis was carried out using the program GARLI v.2.0 [50], using default parameters except where specified. Phylogenetic estimates using ML were performed using both the combined, unpartitioned dataset as well as the combined dataset partitioned by locus (Additional file 2: Table S2). Five ML search replicates were run for each dataset using random starting trees, and up to five million generations were employed for each run unless the scoring topology lnL did not improve by ≥ 0.01 for 20 000 generations, in which case the run was terminated prematurely and the next bootstrap replicate was begun. Two hundred bootstrap replicates were conducted for each run and consensus trees were generated using the SumTrees v.3.0 software which is part of the DendroPy v.3.7 phylogenetic computing library [51]. Likelihood ratio tests (LRTs) [13,52] were performed to compare competing model partitioning schemes, M0 and M1. Statistical support for model M0 over M1 (or vice versa) was assessed using the Chi-square distribution for *q* degrees of freedom (df) where *q* equals the difference in the number of free parameters between model M0 and M1 (df = 19 in this study) [52].

## Results

### Gene fragments used for analyses contain differing degrees of variability

Among the four gene fragments analyzed in this study, 3 521 nucleotides were included (1 481 from the 16S rRNA gene fragment, 816 from *dnaJ*, 474 from *rpoB*, and 750 from *tuf*) for 59 different taxa. The dataset contained 1 016 parsimony-informative sites and 2 142 conserved sites. The nucleotide diversity of the 16S rRNA gene fragment was 0.029 substitutions (subs.) per site, while that for *dnaJ, rpoB,* and *tuf* was 0.241, 0.147, and 0.097 subs. per site, respectively. The average theta per site for the combined dataset was 0.04. The lowest interspecies divergence was between *S. pseudintermedius* and *S. delphini* (0.014 subs. per site). The highest estimated evolutionary divergence within the complete dataset was between *S. piscifermentans* and the outgroup species, *B. subtilis* (0.266 subs. per site), while the highest level among staphylococcal taxa was between *S. piscifermentans* and *S. vitulinus* (0.182 subs. per site).

### Individual gene tree analyses

Phylogenetic analysis of individual genes revealed that 16S rRNA and *dnaJ* fragments resolved similar major clades but different branching orders of these clades (Additional file 3: Figure S1). Similarly, most relationships and clusters of species within *rpoB* and *tuf* gene trees were in general agreement with the 16S and *dnaJ* gene trees, but multiple higher-level clades were present in these that were unique (Additional file 3: Figure S1). Thus, individual gene tree analyses supported similar clusters of species, but varying arrangements of these clusters relative to one another. As expected, nodal support values for individual gene trees were relatively low, particularly for nodes more deeply nested in the tree. Formal partition homogeneity (or incongruence length difference test [29]) tests indicated that there were significant differences between all partitions except for 16S rRNA gene and *dnaJ*. This test, however, is based on parsimony criteria and known to have highly variant type-1 and type-2 error rates depending on different tree structures, rates across sites, and informative site contents among datasets [53,54]. We therefore interpret these results cautiously, as the potential for there to exist some conflicting phylogenetic signal among genes, and incorporate this caution in later interpretations of the combined data analyses. Such conflicting signal might result from multiple sources, including phylogenetic estimation error leading to different inferences from individual gene trees, and/or different underlying evolutionary histories among genes due to lateral gene transfer or lineage sorting effects.

### Dataset partitioning improves likelihood estimates of Bayesian phylogenetic analyses

**Figure 1:**
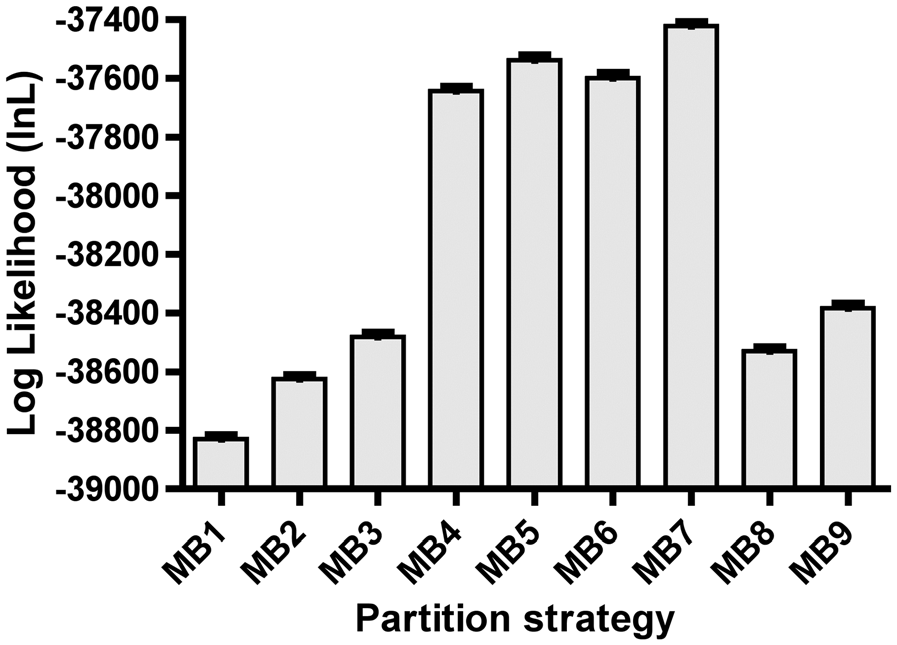
**Dataset partitioning improves model fit.** Shown are log-likelihood plots comparing partitioning strategies used for concatenated BI runs. Error bars represent the mean ± 95% confidence interval.

Regardless of partitioning strategy employed, all Bayesian inference (BI) runs yielded highly reproducible phylogenetic inferences (Additional file 4: Figure S2). Within MrBayes BI runs, log-likelihood (lnL) estimates rapidly reached stationarity and convergence. Log-likelihoods ranged from −38830.66 (MB1) to −37421.36 (MB7) with intermediate lnL generally increasing with partition complexity (Figure 1). Dataset partitioning for concatenated BI runs (i.e., MrBayes) ranged from the most simple (unpartitioned) to highly complex (11 partitions; Table 1). Initial assessments of Bayes factors (BF; 2ΔlnB_10_) were used to compare topological likelihoods across each different model. As shown in Table 2, a large disparity between the lnL from various partitioning strategies was observed. Partitioning strategy MB7 yielded the highest lnL (Figure 1) with a BF > 230 that of the next best model (MB5) and >2800 compared to the unpartitioned model (MB1). Model MB7 was the most complex strategy (11 different partitions) with a separate model for each codon position of each protein-coding gene, as well as stem versus loop regions of the 16S rRNA gene fragment (Table 1). The model with the second highest likelihood was MB5 whereby the 16S rRNA gene fragment was again partitioned by stem and loop position, however, only two independent partitions were applied to each individual protein coding gene fragment (codon positions 1 & 2; and codon position 3). Using AIC_c_ for the Aw calculation identified model MB5 as the best-fit model (Aw = 1.000; Table 2). Thus, based on lnL-centric criteria, models MB5 and MB7 are the preferred models for the concatenated data analysis.

**Table 2:** Bayes factors and Akaike weights reveal differences in model fitness for the different partitioning strategies applied to the concatenated, multilocus dataset

**M1↓**	^**a**^**2ΔlnB**_**10**_									**Akaike Weight**
**M0→**	**MB1**	**MB2**	**MB3**	**MB4**	**MB5**	**MB6**	**MB7**	**MB8**	**MB9**	
MB1	---	**412.11**	**700.90**	**2371.08**	**2584.86**	**2462.04**	**2818.59**	**603.91**	**891.39**	0.000
MB2	−412.11	---	**288.79**	**1958.97**	**2172.74**	**2049.93**	**2406.48**	**191.80**	**479.28**	0.000
MB3	−700.90	−288.79	---	**1670.18**	**1883.96**	**1761.14**	**2117.70**	−96.99	**190.49**	0.000
MB4	−2371.08	−1958.97	−1670.18	---	**213.77**	**90.96**	**447.51**	−1767.17	−1479.69	0.000
MB5	−2584.86	−2172.74	−1883.96	−213.77	---	−122.82	**233.74**	−1980.95	−1693.46	1.000
MB6	−2462.04	−2049.93	−1761.14	−90.96	**122.82**	---	**356.56**	−1858.13	−1570.65	0.000
MB7	−2818.59	−2406.48	−2117.70	−447.51	−233.74	−356.56	---	−2214.68	−1927.20	0.000
MB8	−603.91	−191.80	**96.99**	**1767.17**	**1980.95**	**1858.13**	**2214.68**	---	**287.48**	0.000
MB9	−891.39	−479.28	−190.49	**1479.69**	**1693.46**	**1570.65**	**1927.20**	−287.48	---	0.000

^a^Positive Bayes factors (2ΔlnB_10_) support model M0 over model M1 and negative values support model M1 over model M0. Bayes factor support values >10 are shown in bold.

Inspection of TL identified that the more highly partitioned models (MB4-MB7) yielded TLs between two and four times longer than less partitioned models (MB1-3; MB8-9; refer to Additional file 5: Figures S3 and Additional file 6: Figure S4). The more highly-partitioned model runs with high TLs also tended to show very high TL variance among generations, resulting in quite broad TL posteriors (Additional file 5: Figures S3 and Additional file 6: Figure S4). Considering this evidence for unreliability in the more highly partitioned model runs, we tempered our choice of partitioning scheme. A combination of lnL (BF and Aw) and TL reliability criteria suggest that MB8 is the preferred partitioned model, since it had better lnL than other models (e.g., MB1-2) while resulting TL estimates were apparently uninflated and of low variance (Additional file 5: Figures S3 and Additional file 6: Figure S4). Hereafter, we discuss results based on the BI runs from model MB8, and identify any notable differences between this model and others (particularly MB5 and MB7). It is important to note however that while lnL and TLs differed between partitioning scheme models, tree topologies remained nearly identical (discussed below). It is possible that the inconsistency between the BF-based support for more highly partitioned models versus evidence for model overparameterization that we observed may be related to calculation of BFs based on harmonic mean approximations of marginal likelihoods, which has been shown previously [42,43,45]. Thus, while no substantial topology or support value differences were observed between results from different models, we have taken a conservative approach and chosen to use MB8 as the preferred model because more complex models exhibited excessive TL indicative of overparameterization.

### Bayesian and maximum likelihood analyses of concatenated data

**Figure 2:**
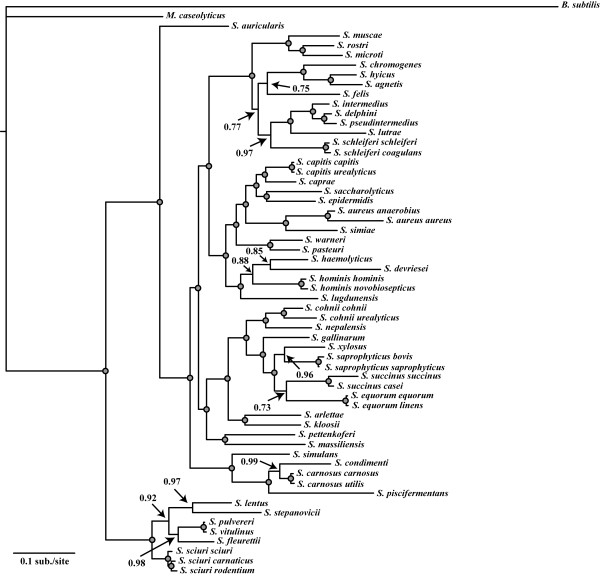
**Bayesian MCMC analysis of the concatenated dataset.** Shown is a 50% majority rule phylogram from BI runs under the combined, partitioned dataset in MrBayes. Numbers represent posterior probabilities with grey-filled circles representing a posterior probability of 1.00.

Regardless of the model under which the concatenated staphylococcal dataset was analyzed using BI, high overall nodal support was observed for nearly all nodes in the tree. Tree topologies were highly concordant between different partitioned model schemes, with only a single topological inconsistency between models. In addition to the placement of *S. devriesei* shown in Figure 2, this species was also estimated to form a clade with *S. lugdunensis* under four models (MB2-4, and MB6). Additionally, under models MB5 and MB7, *S. devriesei* was estimated to diverge after *S. lugdunensis*, forming the sister lineage to a clade containing *S. haemolyticus* and *S. hominis* (data not shown). Nodal support for these alternative relationships was quite low (avg. Pp = ~0.64), however, in comparison to the support of *S. devriesei* forming a clade with *S. haemolyticus* (Pp = 0.85; Figure 2). Beside this single topological difference, nodal support differed by very little among models (Pp ≤ 0.02), with only two cases (MB1 and MB6) in which a single node differed by a Pp = 0.05.

Bayesian concatenated phylogenetic estimates supported strongly (Pp = 1.00) the separation of staphylococcal species into two deeply-diverging major clades (Figure 2). One of the two clades contained all of the oxidase positive staphylococcal species (frequently referred to as the Sciuri group), with the second group containing all other oxidase negative staphylococcal species (Figure 2). The single lineage *S. auricularis* formed the sister group to all other members of this second group, with the next most basally-diverging lineage in this clade including the following species: *S. simulans, S. condimenti, S. carnosus* (both subspecies)*,* and *S. piscifermentans* (Pp = 1.00). The subspecies of *S. carnosus* proved to cluster tightly together, as expected, and formed the sister group to *S. condimenti*.

The next major divergence within the staphylococcal tree was that of a strongly supported clade (Pp = 1.00) containing the pathogenic species *S. saprophyticus* (Figure 2). This clade contained many members of the polyphyletic group of coagulase negative, novobiocin resistant species, and included the recently described species *S. massiliensis*[55] and *S. pettenkoferi*[56]. Following this divergence, species of heightened clinical significance diverged, including *S. aureus, S. epidermidis, S. warneri, S. haemolyticus* and *S. lugdunensis,* which formed a well-supported clade (Pp = 1.00) (Figure 2). We also found that the most recently discovered *Staphylococcus* species, *S. agnetis*[57] formed a strongly supported clade (Pp = 1.00) with *S. hyicus*, for which *S. chromogenes* was the sister lineage.

**Figure 3:**
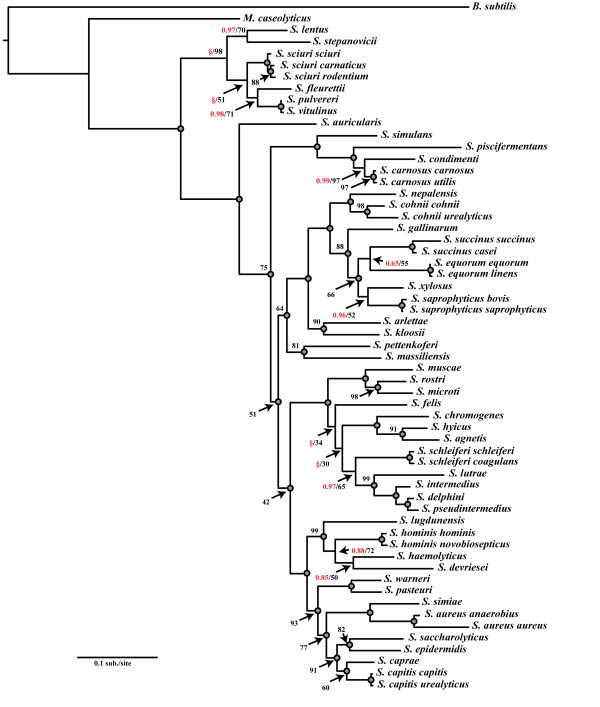
**Maximum likelihood phylogram of staphylococcal species.** Shown is a ML phylogram obtained from the assessment of the locus-partitioned dataset (similar to MB3) using GARLI v.2.0 [50]. The consensus phylogram was generated from 200 bootstrap replicates with five ML search replicates per bootstrap. Nodes receiving Pp = 1.00 and/or BS = 100% are indicated by grey-filled circles; otherwise, MrBayes posterior probability is shown in red text, and ML bootstrap support is shown in black text. Clades that were not present in MrBayes are indicated by a red §.

**Figure 4:**
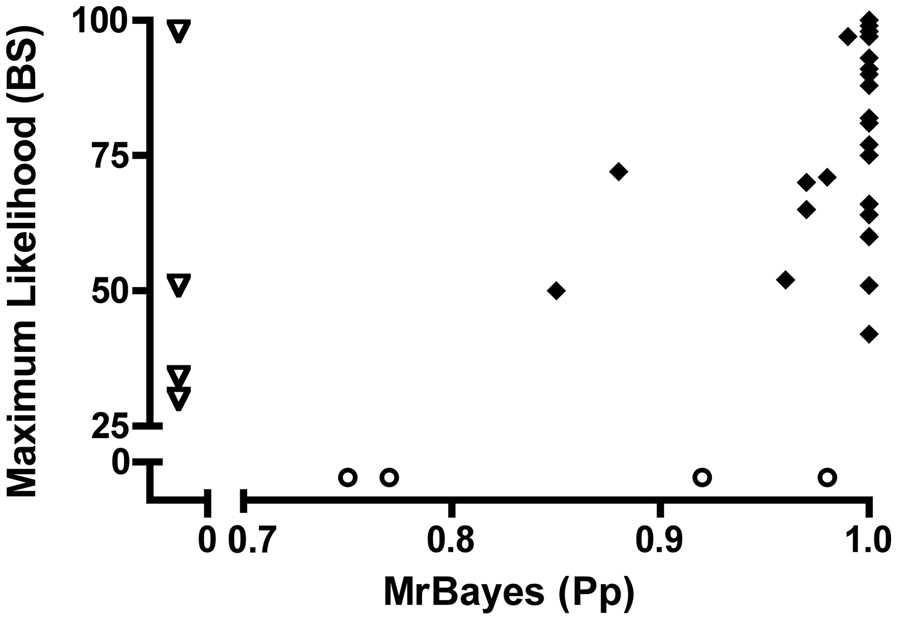
**Comparison of nodal support between MrBayes and maximum likelihood methodologies.** Shown is a scatter plot comparing the differences in MrBayes posterior probabilities (Pp) and maximum likelihood (ML) bootstrap support (BS) for identical nodes (Figure 3). Open circles represent Pp support for discordant nodes present in MrBayes and absent in ML. Open triangles represent BS values for discordant nodes present in ML and absent in MrBayes. Note that MrBayes exhibits heightened overall node support as compared to ML.

The concatenated ML estimation of the staphylococcal phylogeny was consistent with reconstructions from the concatenated BI method (Figure 3). Log-likelihoods under a single evolutionary model were −39186.39 while partitioning the concatenated dataset by individual gene yielded a lnL = −36632.34. The likelihood-ratio test supported the partitioned dataset as the best-fit model (p < 0.0001; likelihood-ratio (−2ΔlnL) = 5 108; degrees of freedom (df) = 19). Topologies estimated under both models were identical except for a single discordant node: *S. devriesei* formed a single-species sister taxon to *S. haemolyticus* and *S. hominis* in the unpartitioned dataset (bootstrap support (BS) = 59%), while in the dataset partitioned by individual gene, *S. devriesei* shared a clade with *S. haemolyticus* (BS = 72%). Aside from minor discrepancies between the concatenated ML and BI topologies (Figure 3), high topological agreement was observed. Although weakly supported (BS < 50%), *S. felis* diverged more deeply under ML than BI, forming a single species sister lineage to the larger clade containing *S. hyicus, S. intermedius*, and *S. schleiferi* in the ML tree (Figure 3). Among the oxidase containing species clade, ML estimated a more basal divergence of *S. lentus* and *S. stepanovicii* than was estimated under the concatenated BI approach (Figure 3). Comparisons between BI and ML nodal support values indicated that support values at discordant nodes between BI and ML methods ranged from Pp = 0.75-0.98 for BI and BS = 30-98% for ML (Figure 4). Thus, differences in the tree and support values between methods included both weakly and strongly supported nodes.

### Concatenated and unconcatenated phylogenetic methods broadly agree on clustering of staphylococcal species

Estimation of staphylococcal phylogeny was also performed on the unconcatenated dataset using Bayesian Estimation of Species Trees (BEST) analysis [36]. BEST analyses treated each locus as an independent gene, thereby inferring the likely species tree given four independent gene trees. Trees inferred from duplicate BEST runs were identical in topology with no nodes differing by Pp > 0.05, indicating that multiple runs converged on nearly the same posterior tree space. With three exceptions, the BEST tree resolved the same major clades as the concatenated BI and ML trees, although in some cases the relative branching order of major clades differed (Additional file 7: Figure S5). BEST estimated that *S. auricularis* formed a clade with *S. sciuri* (Pp = 0.99) as opposed to the later (less basal) divergence of *S. auricularis* observed in concatenated BI and ML estimates (Pp = 1.00 and BS = 100%, respectively; Additional file 7: Figure S5). BEST also estimated a more divergent relationship between *S. kloosii* and *S. arlettae,* whereas these two species formed an exclusive clade in concatenated data analyses. The concatenated BI and ML analyses estimated *S. felis* to diverge more basally than was inferred by BEST analyses, although support for the placement of *S. felis* was generally low among all methods (BEST, Pp < 0.50; concatenated BI, Pp = 0.75; concatenated ML, BS < 50%).

In order to achieve convergence using BEST method, we chose to enforce a molecular clock to reduce the number of parameters in the analysis. To evaluate the impact of this parametric restriction on the resulting inferences, we also conducted BEST analyses without enforcing a molecular clock. Only minor differences in cluster groupings between analyses were observed where *S. agnetis* and *S. hyicus* formed a clade with *S. chromogenes* as the sister taxon in Additional file 7: Figure S5, while the alternate (non-clock BEST) analysis estimated *S. agnetis* and *S. chromogenes* to form a clade, sister to *S. hyicus* (data not shown). However, because convergence was not achievable without application of a strict molecular clock, overall node supports for this tree tended to be lower than the clock-constrained BEST analysis. These results also suggest that differences in tree topology between concatenated methods and BEST analyses are not necessarily the result of applying a molecular clock to the dataset.

**Figure 5:**
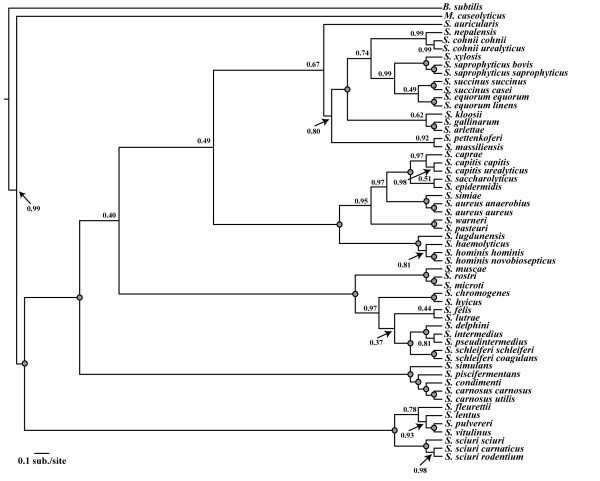
**Inference of the staphylococcal phylogeny using Bayesian estimation of species trees (BEST) methodology on 16S rRNA and*dnaJ*gene fragments.** Shown is a consensus phylogram of the staphylococcal species tree generated under the BEST methodology incorporating only 16S rRNA and *dnaJ* gene fragments. Each of the two gene fragments were treated as an individual locus for which individual gene trees were estimated. Numbers represent posterior probabilities with grey-filled circles representing a posterior probability of 1.00. Refer to Additional file 7: Figure S5 for the BEST analysis incorporating all four gene fragments.

To obtain a more robust estimate of the staphylococcal phylogeny using BEST, additional datasets were assessed in which suspected conflicting loci and taxa were omitted. Omission of the *tuf* and *rpoB* loci, as well as taxa for which data were missing (i.e., *S. agnetis, S. stepanovicii,* and *S. devriesei*), substantially altered the branching order of major clades (as compared to original BEST methodologies incorporating all gene fragments and taxa), resulting in higher agreement with the concatenated BI and ML analyses (Figure 5). These data also support our previous ILD tests that suggested a significant difference between all loci except 16S rRNA and *dnaJ* (above). With the exception of a few notable differences, the modified BEST analysis was similar to the concatenated BI and ML analyses (Figure 5). The modified BEST analysis estimated, with weak support, the later divergence of *S. auricularis* as compared to BI and ML runs. This analysis also estimated a more basal divergence of the clades containing *S. muscae, S. hyicus,* and *S. intermedius* with a later divergence of clades containing *S. pettenkoferi, S. arlettae, S. saprophyticus,* and *S. lugdunensis* as compared to concatenated analyses (Figure 5). *S. felis* and *S. lutrae* shared a weakly supported clade (Pp = 0.44) within this BEST analysis as compared to belonging to different clades (described above) in concatenated BI and ML data analyses. *S. gallinarum* formed a clade (Pp = 1.00) in the modified BEST analysis with *S. arlettae,* and *S. kloosii* while concatenated analyses estimated *S. arlettae* to form an exclusive clade with *S. kloosii* and *S. gallinarum* belonging to a more distant clade (compare Figures 3 and 5).

## Discussion

### Using multilocus data to infer the *Staphylococcus* phylogeny

*Staphylococcus* is a species-rich genus of importance from both a human health and economic perspective. Relevant to the first goal of this study, our results provide strong evidence that the current groupings of *Staphylococcus* species require revision, and provide a clear consensus across analyses on how this could be done to reflect inferred evolutionary relationships among species groups. The second goal of the study was to infer higher-level relationships among species and cluster groups, and our results provide good evidence for much consensus across methods although there remains some alternative hypotheses for such higher-level relationships that differed between methods. To infer phylogenies relevant to both species-grouping and higher-level relationships, we used a combination of Bayesian and maximum likelihood analyses of multilocus data. We found that both Bayesian and maximum likelihood analysis of multilocus data yielded high-resolution species trees with overall high nodal support values for relationships among *Staphylococcus* species. We also found that partitioned-model analysis of the combined dataset, versus the concatenation-free analysis using BEST, produced near-identical estimates of the species composition of major clades and putative revised cluster groupings (i.e., more recent relationships).

In contrast to broad consensus across methods for resolution of relationships among more recent groupings of species, concatenated and gene-tree-based methods (BEST) inferred several alternative relationships among more ancient lineages of staphylococcal species. It is not entirely clear, however, what the source of these differences are (e.g., different evolutionary histories among genes being differentially resolved between methods, difference in how methods extracted signal from the multilocus data, etc.). It is notable that our finding that the BEST method inferred similar major clades as the concatenated methods, but inferred a different branching order among these major clades, has also been observed in other studies [40]. Such an observation may be indicative that although the phylogenetic signal contained within gene trees affords robust estimates of membership of particular species to major clades, conflicting signal or simply very little signal for deeper relationships among major lineages is available from single gene tree inferences (as in BEST). Our analysis of individual gene trees further supports this hypothesis whereby there appears to be substantial disagreement about higher-level relationships, but individual gene trees are consistent with one another regarding the placement of species within clusters towards the tips of the tree. It is notable that the modified BEST analysis, in which only two gene fragments were incorporated, more consistently resolved higher order relationships with the concatenated BI and ML methodologies. This suggests further that conflicting signal within the other two gene fragments was contributing to the discord among the original BEST analysis, incorporating all four loci.

It has been shown that staphylococcal species routinely laterally transfer genes [58], and it is therefore reasonable to consider that lateral gene transfer might complicate inference of phylogeny in this study. For example, lateral transfer (potentially combined with phylogenetic inference error) may explain instances of disagreement between gene trees and multi-locus inferences. Particularly in the case of inferring bacterial phylogeny, generally high instances of gene transfer inherently complicate inference of species-level trees, and even raise philosophical questions about the meaning of such species-level inferences [59]. Our results do, however, provide good evidence that there is indeed phylogenetic signal of an underlying species-level tree with many shared relationships across analytical methods, and this tree contrasts strongly with the existing higher-level classification scheme of the group that was based on less robust inferences methods. Our results largely agree across methods about the membership of species and subspecies to major clades, and thus provide new important confirmatory information sufficient for refining the nomenclature of the group.

**Figure 6:**
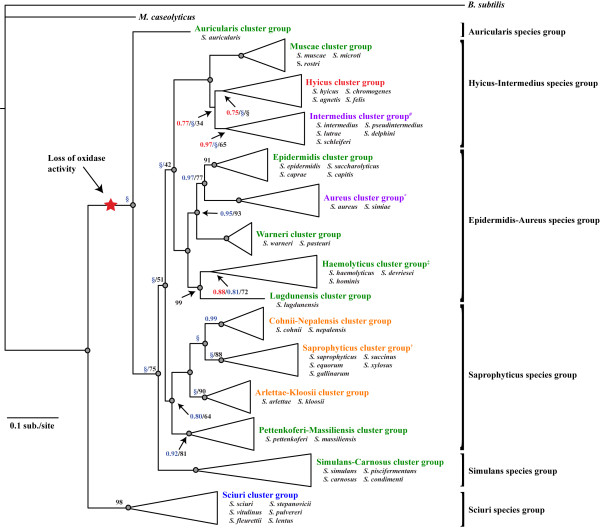
**Staphylococcal species can be combined into six species groups and 15 cluster groups.** Shown is a summary phylogram adapted from Figure 2 with clades collapsed to represent staphylococcal groupings. Whenever possible, cluster and species group names were kept consistent with [64]. Cluster groups have been color-coded to represent: **blue**, species that are novobiocin resistant, coagulase negative, and oxidase positive; **green**, species that are novobiocin susceptible, coagulase negative, and oxidase negative; **orange**, species that are novobiocin resistant, coagulase negative, and oxidase negative; **purple**, species that are novobiocin susceptible, coagulase positive, and oxidase negative; and **red**, species that are novobiocin susceptible, coagulase variable, and oxidase negative. Color scheme exceptions are: ^#^*S. schleiferi schleiferi* is coagulase negative; *S. simiae is coagulase negative; ^‡^*S. hominis novobiosepticus* is novobiocin resistant; and ^†^*S. equorum linens* is novobiocin susceptible. Members of each cluster group are listed below the cluster group name. Nodes receiving Pp = 1.00 or BS = 100% are indicated by grey-filled circles; otherwise, MrBayes posterior probability is shown in red text, BEST posterior probability is shown in blue text, and ML bootstrap support is shown in black text. Clades that were not present in BEST or ML are indicated by a blue or black §, respectively.

Historically, staphylococcal species have been clustered into between four and eleven species groups [6,60-64]. Most of these groupings, however, were inferred based on a single locus with a small number of staphylococcal taxa. Phylogenetic estimates from this study suggest the separation of staphylococcal species into six major staphylococcal species groups comprised of 15 refined cluster groups (Figure 6). We have used our Bayesian, partitioned-model concatenated data estimate (i.e., Figure 2) as the focal phylogeny for illustrating evolutionary groupings of *Staphylococcus* since this phylogeny was also supported by our ML analysis, and previous reports on phylogenetic estimates of the staphylococcal phylogeny. Additionally, this phylogeny was essentially the same regarding these cluster groups based on the BEST tree. Current knowledge of phenotypic properties and relationships among staphylococci are also in agreement with the staphylococcal phylogeny estimated from concatenated analyses. For example, concatenated analyses resolved the oxidase positive species as being the sister to the remaining species, which is sensible given that outgroups of staphylococcal species are also oxidase positive; this relationship was also observed in the modified BEST analyses. For the purposes of reference, we indicate on the concatenated BI tree (Figure 6) where ML concatenated and BEST inferences differed. Wherever possible, we have attempted to name cluster groups and species groups following the original nomenclature put forth by Takahashi *et al*. [64], while recognizing only evolutionarily distinct, monophyletic groupings based on our estimates of phylogeny. As with previous analyses, cluster groups represent a single monophyletic clade of species, based on branching pattern [64], while species groups follow those previously described by Kloos *et al.*[65] and present cluster groups sharing similar phenotypic properties [64,66].

### Refined phylogeny and classification of *Staphylococcus* spp.

Consistent with previous studies [55,63,64,67,68], our analyses identified the monophyletic group containing the novobiocin-resistant, oxidase positive species (Sciuri group; Figure 6, blue cluster group) as the sister group to all other *Staphylococcus*. This cluster group also contains the recently discovered species, *S. stepanovicii*[11]. Within this group, we inferred a close relationship, with little sequence divergence, between *S. vitulinus* and *S. pulvereri* (BI and BEST Pp = 1.00; BS = 100%), potentially supporting the reclassification of *S. pulvereri* as a later synonym of *S. vitulinus*[67]. After the basal divergence of the Sciuri group, the second lineage to diverge from the remaining staphylococcal lineages was the oxidase negative Auricularis group, containing only *S. auricularis* (Figure 6). Our phylogeny therefore suggests that cytochrome C oxidase was lost in *Staphylococcus* sometime in the common ancestor of *S. auricularis* and the remaining *Staphylococcus* species, after their divergence from the Sciuri group (Figure 6, red star).

Our phylogenetic placement of *S. auricularis* as the sister lineage to all non-Sciuri group staphylococci is unique to our study, and we find strong support for this inference (MrBayes Pp = 1.00 and ML BS = 99%). Based on the 16S rRNA gene alone, Takahashi *et al.*[64] estimated that *S. auricularis* shared a common ancestor with the *S. saprophyticus, S. lugdunensis, S. haemolyticus, S. warneri, S. epidermidis* and *S. aureus* cluster groups. More recently, Ghebremedhin *et al.*[6] estimated a similar relationship to that of Takahashi *et al.* based on 16S rRNA gene alone. Analyses of subsequent gene fragments, however, yielded varying relationship estimates for *S. auricularis,* and no previous studies have found particularly strong support for the placement of this lineage. For example, Ghebremedhin *et al.*[6] recovered bootstrap support of 31% for a clade containing *S. auricularis* and *S. kloosii* based on the 16S rRNA gene, although average BS support across their tree was particularly low, at BS = 52%. Similarly, *S. auricularis* was placed as the sister lineage to *S. kloosii* plus the *S. saprophyticus* group, with BS = 25% based on analysis of the 16S rRNA gene by Takahashi *et al.*[64].

We inferred that the next lineage of *Staphylococcus* to diverge was the Simulans species group (Figure 6), which contains four species that are all novobiocin susceptible and coagulase negative. For consistency with previous nomenclature [6,64], we refer to this clade as the Simulans-Carnosus cluster group and the species group as the Simulans group (Figure 6). Our estimate of relationships among species of this group agree with previous studies, although the inclusion of *S. condimenti* in our trees is novel [6,64]. We inferred a single clade (Simulans-Carnosus cluster) containing the novobiocin susceptible, coagulase negative species, *S. simulans, S. condimenti, S. carnosus* and *S. piscifermentans.*

Following the split of these three early-diverging lineages, the remaining *Staphylococcus* species diverged into three large species groups. The first of these to diverge from the remaining was the Saprophyticus species group (Figure 6), which we inferred consists of four cluster groups. Within this species group, the Pettenkoferi-Massiliensis cluster group contains novobiocin susceptible species while all of the remaining members of the Saprophyticus group are novobiocin resistant. Thus, it seems that an alternative gyrase B gene conferring novobiocin resistance may have been acquired in this clade sometime after the Pettenkoferi-Massiliensis cluster group diverged from the rest of the Saprophyticus species group. Based on analysis of the 16S rRNA gene, Al Masalma *et al.*[55] reported the newly discovered species *S. massiliensis* to be a member of the Simulans group, although they failed to recover this relationship in analyses of the *dnaJ, rpoB,* and *tuf* genes, where they instead placed it with *S. pettenkoferi* as we have here. It is also notable that the close relationship between these coagulase-negative species was also suggested based on their phenotypic similarities across a range of biochemical tests [55]. Additionally, in the Saprophyticus cluster group, we inferred a close relationship between *S. equorum, S. succinus, S. saprophyticus,* and *S. xylosus* with *S. gallinarum* as the sister lineage to these four species. The placement of *S. gallinarum* in other studies is variable, but on multiple occasions has clustered with the Arlettae-Kloosii group [6,57,60,63,64]. This alternative placement of *S. gallinarum* seems reasonable as we find the Arlettae-Kloosii cluster group to be closely related to the Saprophyticus cluster group (Figure 6).

The Epidermidis-Aureus species group contained five cluster groups, including the most common taxa of heightened clinical significance [6]. In general, our estimates of relationships among these species are consistent with previous reconstructions [57,64]. Relationships within the Haemolyticus cluster group also agree with previous estimates [64], with the placement of the recently discovered coagulase-negative bovine strain, *S. devriesei,* forming a clade with *S. haemolyticus*[69]. Lastly, the Hyicus-Intermedius species group contained species of the "*S. hyicus-S. intermedius* cluster group" originally proposed by Takahashi *et al*. [64] based on a 16S rRNA gene dataset, and additional studies have found similar estimates of relationships based on analyses of other loci [1,6,60,61,63,70]. The limited number of taxa assessed in these studies has, however, prevented a more detailed understanding of species relationships within this species group prior to our analysis here. Moreover, recent novel species discovery (in particular *S. rostri*[70]*, S. microti*[1]*,* and *S. agnetis*[57]) has also contributed to the enhanced diversity of the Hyicus-Intermedius group. We have divided this species group into three cluster groups based on their phylogenetic relationships, which is also supported by their phenotypic diversities (Figure 6). Species among the Intermedius cluster group are all coagulase positive, excepting *S. schleiferi schleiferi*. Interestingly, *S. schleiferi coagulans* is coagulase positive, consistent with the other members of this cluster group, implying a recent loss in *S. schleiferi schleiferi*. In contrast, the Muscae cluster group contains only coagulase negative species (*S. muscae, S. rostri,* and *S. microti*). Within the last two years, both *S. rostri*[70] and *S. microti*[1] were discovered and found to cluster with *S. muscae,* thus altering previously known relationships within this species group. The Hyicus cluster group is coagulase-variable, including coagulase positive (*S. hyicus*), negative (*S. chromogenes, S. felis*), and variable (*S. agnetis*) species (Figure 6, red cluster group).

## Conclusions

Through the analysis of multiple loci under a variety of phylogenetic methods, we achieved one of our main goals of inferring a robust estimate of the cluster groupings among staphylococcal species. We have used this estimate of cluster groupings to refine the current knowledge of the systematics and nomenclature for this important genus. Our results also contribute to a presumably more accurate understanding of the higher-level relationships among *Staphylococcus* species, although we do note that there are several outstanding questions left by the alternative resolutions of our concatenated versus species-tree-based inferences. We have attempted to present these yet unresolved inferences in a transparent fashion such that future work might directly test remaining alternative hypotheses and add further clarity to the relatively small number of remaining questions about relationships among staphylococcal species. The availability of such a comprehensive estimate of the evolutionary origins of, and relationships among, staphylococci provides an important context for understanding patterns of gain and loss of genetic and physiological attributes, and the potential role of lateral gene transfer in both pathologically-relevant phenotypes and in estimation of phylogenetic relationships among species. Such questions are of particular relevance considering the clinical and economical significance of some *Staphylococcus* species. Approaches such as this will provide a more natural classification of species based on phylogenetic inferences and lend support to future evolutionarily-informed studies of microbial diversity and physiology.

## Competing interests

The authors declare that they have no competing interests.

## Authors’ contributions

RPL participated in study conception and design, data acquisition, genetic analyses, and manuscript preparation. GM participated in data acquisition, maximum likelihood analyses, and manuscript preparation. TAC contributed to conceptualization, data interpretation, and preparation of the manuscript. ST participated in data acquisition, and manuscript revision. AMC provided analytical software and hardware, participated in study design, data analysis, and manuscript preparation. CLP provided analytical software, participated in study design, data analysis, and manuscript preparation. All authors read and approved the final manuscript.

## Supplementary Material

Additional file 1: Table S1 **GenBank accession numbers for 16S rRNA gene fragments,*dnaJ, rpoB,* and*tuf*gene fragments analyzed in this study.** Click here for file

Additional file 2: Table S2 Evolutionary models for each partition were chosen based on AIC using jModelTest. Click here for file

Additional file 3: Figure S1 **Gene trees for individual loci assessed in this study.** Shown are Bayesian 50% majority rule phylograms for A) the 16S rRNA, B) *dnaJ,* C) *rpoB,* and D) *tuf* gene fragments. MrBayes was run under the same conditions as those used for concatenated analyses with evolutionary model specified for whole gene fragments in Additional file 2: Table S2. Numbers represent posterior probabilities with grey-filled circles representing a posterior support of 1.00. Click here for file

Additional file 4: Figure S2 **Bayesian inferences of phylogeny are highly reproducible, regardless of model employed.** Shown are plots of post-burnin generational log likelihoods (lnL) from five representative partitioning strategies across triplicate concatenated BI runs (A); and duplicate BEST runs (B). All runs were highly reproducible regardless of methodology and partitioning strategy. Click here for file

Additional file 5: Figure S3 **Tree length (TL) analysis indicates that overparameterization may be occurring within more highly partitioned datasets.** Shown are post-burnin generational TL estimates for partitioning strategies assessed in this study. Note that as the complexity of partitioning increases evidence of increased TL and failed convergence is observed. Click here for file

Additional file 6: Figure S4 **Model partitioning increases the mean tree length (TL) and run variance.** Shown is a box plot indicating the mean TL and 95% confidence interval among partitioning strategies. Click here for file

Additional file 7: Figure S5 **Inference of phylogeny using Bayesian estimation of species trees (BEST).** Shown is a consensus phylogram of the staphylococcal species tree generated using all four gene fragments under the BEST methodology. Each gene fragment was treated as an individual locus for which individual gene trees were estimated (similar to MB3). Numbers represent posterior probabilities with grey-filled circles representing a posterior probability of 1.00. Click here for file
