# Supplementary material for: Phylogenetic relationships among Staphylococcus species and refinement of cluster groups based on multilocus data
Source: BMC Evol Biol. 2012 Sep 6;12:171. doi: 10.1186/1471-2148-12-171 (PMC3464590; doi:10.1186/1471-2148-12-171)
Supplement: Additional file 1: Table S1 — GenBank accession numbers for 16S rRNA gene fragments,dnaJ, rpoB, andtufgene fragments analyzed in this study. [file 1471-2148-12-171-S1.pdf]

Supplementary Table S1. GenBank accession numbers for 16S rDNA, *dnaJ, rpoB,* and *tuf* gene fragments analyzed in this study.

No.	*Staphylococcus* Species	Subspecies	[a]Strain	[b]Genes			
				16S	*dnaJ*	*rpoB*	*tuf*
1	*S. agnetis*		DSM 23656	HM484980.1	N/A	HM484993.1	HM485006.1
2	*S. arlettae*		ATCC 43957	AB009933.1	AB234056.1	AF325874.1	EU652781.1
3	*S. aureus*	*aureus*	ATCC 12600	D83357.1	AB234058.1	N/A	AB472826.1
4		*anaerobius*	ATCC 35844	D83355.1	AB234057.1	AF325894.1	HM352930.1
5	*S. auricularis*		ATCC 33753	D83358.1	AB234059.1	AF325889.1	EU652784.1
6	*S. capitis*	*capitis*	ATCC 27840	L37599.1	AB234060.1	AF325885.1	AF298798.1
7		*urealyticus*	ATCC 43926	AB009937.1	AB234061.1	DQ120729.1	EU652786.1
8	*S. caprae*		ATCC 35538	AB009935.1	AB234062.1	AF325896.1	EU652787.1
9	*S. carnosus*	*carnosus*	ATCC 51365	AB009934.1	AB234063.1	AF325880.1	EU652788.1
10		*utilis*	DSM 11676	AB233329.1	AB234064.1	DQ120730.1	EU652789.1
11	*S. chromogenes*		ATCC 43764	D83360.1	AB234065.1	AF325892.1	EU652790.1
12	*S. cohnii*	*cohnii*	ATCC 29974	D83361.1	AB234066.1	AF325893.1	EU652791.1
13		*urealyticus*	ATCC 49330	AB009936.1	AB234067.1	DQ120732.1	HM352939.1
14	*S. condimenti*		DSM 11674	Y15750.1	AB234068.1	DQ120733.1	EU652792.1
15	*S. delphini*		ATCC 49171	AB009938.1	AB234319.1	DQ120735.1	EU157611.1
16	*S. devriesei*		CCUG 58238	FJ389206.1	FJ907454.1	FJ389232.1	FJ389248.1
17	*S. epidermidis*		ATCC 14990	D83363.1	AB234069.1	AF325872.1	AF298800.1
18	*S. equorum*	*equorum*	ATCC 43958	AB009939.1	AB234070.1	AF325882.1	EU652795.1
19		*linens*	DSM 15097	AF527483.1	EU652838.1	DQ120736.1	EU652796.1
20	*S. felis*		ATCC 49168	D83364.1	AB234071.1	AF325878.1	EU652797.1
21	*S. fleurettii*		ATCC BAA274	AB233330.1	AB234072.1	DQ120737.1	HM352961.1
22	*S. gallinarum*		ATCC 35539	D83366.1	AB234073.1	AF325890.1	EU652799.1
23	*S. haemolyticus*		ATCC 29970	L37600.1	AB234074.1	AF325888.1	HM352923.1
24	*S. hominis*	*hominis*	ATCC 27844	L37601.1	AB234075.1	AF325875.1	EU652801.1
25		*novobiosepticus*	ATCC 700236	AB233326.1	AB234076.1	DQ120738.1	EU652802.1
26	*S. hyicus*		ATCC 11249	D83368.1	AB234077.1	AF325876.1	EU571080.1
27	*S. intermedius*		ATCC 29663	D83369.1	AB234078.1	AF325869.1	EU652804.1
28	*S. kloosii*		ATCC 43959	AB009940.1	AB234079.1	AF325891.1	EU652813.1
29	*S. lentus*		ATCC 29070	D83370.1	AB234080.1	AY036973.1	HM352944.1
30	*S. lugdunensis*		ATCC 43809	AB009941.1	AB234081.1	AF325870.1	AF298803.1
31	*S. lutrae*		ATCC 700373	AB233333.1	AB234082.1	DQ120739.1	EU652806.1
32	*S. massiliensis*		CCUG 55927	EU707796.1	EU652841.1	N/A	EU652827.1
33	*S. microti*		DSM 22147	EU888120.1	FN433124.1	EU888121.1	N/A
34	*S. muscae*		ATCC 49910	S83566.1	AB234083.1	AF325884.1	EU652807.1
35	*S. nepalensis*		DSM 15150	AJ517414.1	GQ222247.1	GQ222237.1	EU652808.1
36	*S. pasteuri*		ATCC 51129	AB009944.1	AB234084.1	DQ120742.1	EU652809.1
37	*S. pettenkoferi*		DSM 19554	DQ538517.1	EU652829.1	DQ120744.1	EU652810.1
38	*S. piscifermentans*		ATCC 51136	AF041359.1	AB234085.1	DQ120745.1	HM352955.1
39	*S. pseudintermedius*		CCUG 22219	AJ780976.1	EU652840.1	AM921786.1	EU157680.1
40	[c]*S. pulvereri*		ATCC 51698	AB009942.1	AB234086.1	AF325879.1	N/A
41	*S. rostri*		DSM 21968	FM242137.1	FM244714.1	FM242139.1	N/A
42	*S. saccharolyticus*		ATCC 14953	L37602.1	AB234087.1	AF325871.1	EU652814.1

43	*S. saprophyticus*	*bovis*	DSM 18669	AB233327.1	AB234088.1	DQ120746.1	HM352934.1
44		*saprophyticus*	ATCC 15305	D83371.2	AB234089.1	EF173662.1	EU571085.1
45	*S. schleiferi*	*schleiferi*	ATCC 43808	D83372.1	AB234321.1	AF325886.1	EU652818.1
46		*coagulans*	ATCC 49545	AB009945.1	AB234320.1	DQ120747.1	EU571086.1
47	*S. sciuri*	*carnaticus*	ATCC 700058	AB233331.1	AB234322.1	DQ120748.1	EU652819.1
48		*rodentium*	ATCC 700061	AB233332.1	AB234323.1	DQ120749.1	EU652820.1
49		*sciuri*	ATCC 29062	AJ421446.1	AB234324.1	HM146323.1	HM352947.1
50	*S. simiae*		DSM 17636	AY727530.2	GQ222248.1	EU888127.1	HM352931.1
51	*S. simulans*		ATCC 27848	D83373.1	AB234325.1	AF325877.1	EU571090.1
52	*S. stepanovicii*		CCM 7717	GQ222244.1	GQ222254.1	FJ906724.1	N/A
53	*S. succinus*	*succinus*	ATCC 700337	AF004220.1	AB234326.1	DQ120751.1	EU652824.1
54		*casei*	DSM 15096	AJ320272.1	EU652830.1	DQ120750.1	EU652823.1
55	*S. vitulinus*		ATCC 51145	AB009946.1	AB234327.1	DQ120752.1	EU652825.1
56	*S. warneri*		ATCC 27836	L37603.1	AB234328.1	AF325887.1	AF298806.1
57	*S. xylosus*		ATCC 29971	D83374.1	AB234329.1	AF325883.1	HM352950.1

[a]All are type strains; DSM, German Collection of Microorganisms and Cell Cultures; ATCC, American Type Culture Collection; CCUG, Culture Collection, University of Gothenburg; CCM, Czechoslovak Collection of Microorganisms.
[b]N/A, no sequence analyzed. Numbers indicated are GenBank accession numbers.
[c]Reclassified as a later synonym of *S. vitulinus* [1].

Reference:

1. Svec P, Vancanneyt M, Sedlacek I, Engelbeen K, Stetina V, Swings J, Petras P: **Reclassification of *Staphylococcus pulvereri* Zakrzewska-Czerwinska *et al.* 1995 as a later synonym of *Staphylococcus vitulinus* Webster *et al.* 1994.** *Int J Syst Evol Microbiol* 2004, **54:**2213-2215.
