# Supplementary material for: Phylogenetic relationships among Staphylococcus species and refinement of cluster groups based on multilocus data
Source: BMC Evol Biol. 2012 Sep 6;12:171. doi: 10.1186/1471-2148-12-171 (PMC3464590; doi:10.1186/1471-2148-12-171)
Supplement: Additional file 2: Table S2 — Evolutionary models for each partition were chosen based on AIC using jModelTest. [file 1471-2148-12-171-S2.pdf]

Supplementary Table S2. Evolutionary models for each partition were chosen based on AIC using jModelTest.

Partition	Model
All gene fragments	GTR + ΓI
16S, whole fragment	K80 + ΓI
16S, stem nucleotides	SYM + ΓI
16S, loop nucleotides	GTR + ΓI
All protein coding gene fragments	GTR + Γ
dnaJ, whole fragment	GTR + ΓI
dnaJ, codon positions 1 and 2	GTR + Γ
dnaJ, codon position 1	SYM + Γ
dnaJ, codon position 2	HKY + ΓI
dnaJ, codon position 3	GTR + Γ
rpoB, , whole fragment	SYM + ΓI
rpoB, codon positions 1 and 2	SYM + ΓI
rpoB, codon position 1	GTR + ΓI
rpoB, codon position 2	SYM + ΓI
rpoB, codon position 3	GTR + ΓI
tuf, whole fragment	GTR + Γ
tuf, codon positions 1 and 2	JC + Γ
tuf, codon position 1	GTR + Γ
tuf, codon position 2	GTR + Γ
tuf, codon position 3	GTR + Γ
