# Supplementary material for: Phylogenetic relationships among Staphylococcus species and refinement of cluster groups based on multilocus data
Source: BMC Evol Biol. 2012 Sep 6;12:171. doi: 10.1186/1471-2148-12-171 (PMC3464590; doi:10.1186/1471-2148-12-171)
Supplement: Additional file 3: Figure S1 — Gene trees for individual loci assessed in this study. Shown are Bayesian 50% majority rule phylograms for A) the 16S rRNA, B) dnaJ, C) rpoB, and D) tuf gene fragments. MrBayes was run under the same conditions as those used for concatenated analyses with evolutionary model specified for whole gene fragments in Additional file 2: Table S2. Numbers represent posterior probabilities with grey-filled circles representing a posterior support of 1.00. [file 1471-2148-12-171-S3.pdf]

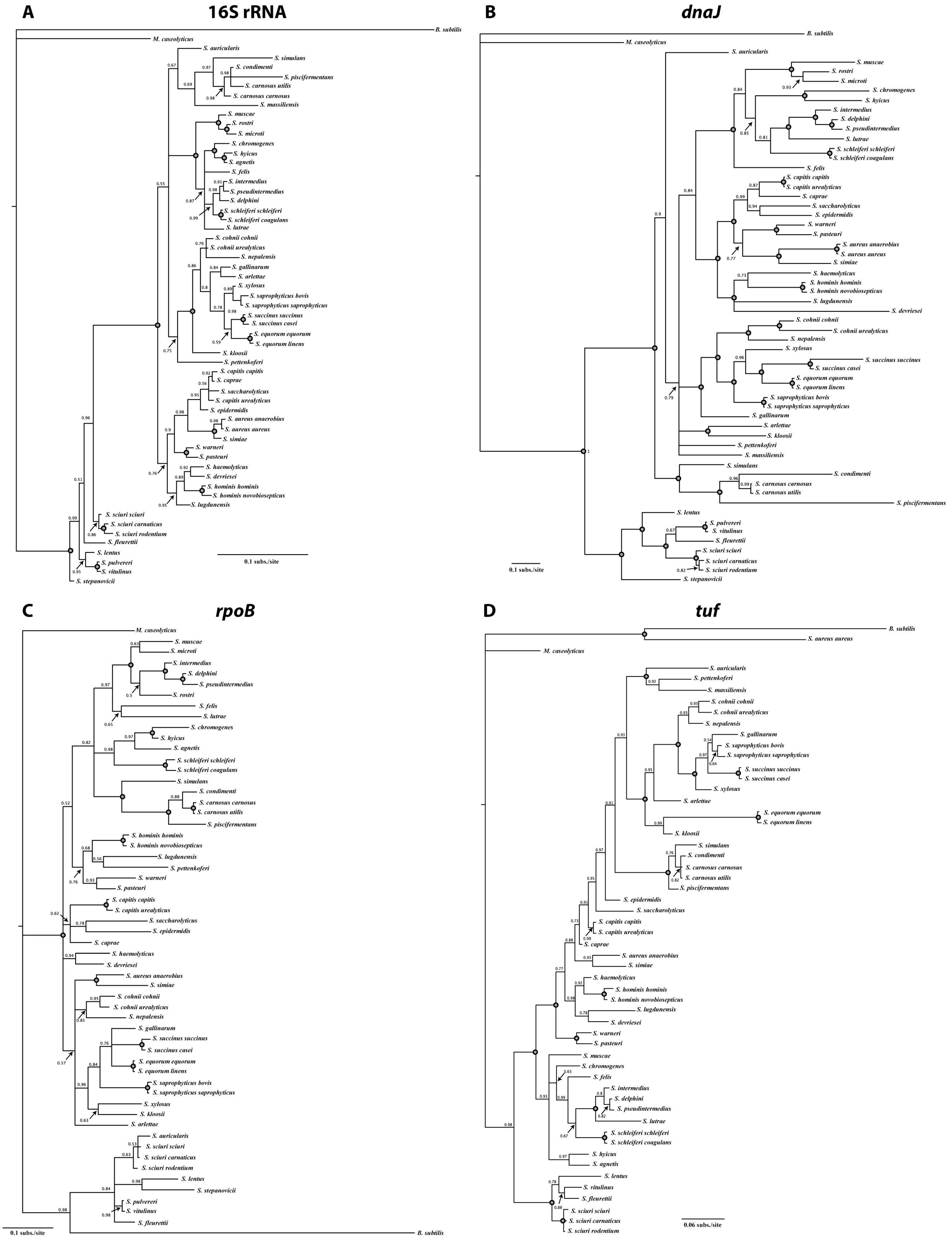
A
16S rRNA
B. subtilis
M. caseolyticus
S. auricularis
S. simulans
S. condimenti
S. piscifermentans
S. carnosus utilis
S. carnosus carnosus
S. massiliensis
S. muscae
S. rostri
S. microti
S. chromogenes
S. hyicus
S. agnetis
S. felis
S. intermedius
S. pseudintermedius
S. delphini
S. schleiferi schleiferi
S. schleiferi coagulans
S. lutrae
S. cohnii cohnii
S. cohnii urealyticus
S. nepalensis
S. gallinarum
S. arlettae
S. xylosus
S. saprophyticus bovis
S. saprophyticus saprophyticus
S. succinus succinus
S. succinus casei
S. equorum equorum
S. equorum linens
S. kloosii
S. pettenkoferi
S. capitis capitis
S. caprae
S. saccharolyticus
S. capitis urealyticus
S. epidermidis
S. aureus anaerobius
S. aureus aureus
S. simiae
S. warneri
S. pasteuri
S. haemolyticus
S. devriesei
S. hominis hominis
S. hominis novobiosepticus
S. lugdunensis
S. sciuri sciuri
S. sciuri carnaticus
S. sciuri rodentium
S. fleurettii
S. lentus
S. pulvereri
S. vitulinus
S. stepanovicii
0.1 subs./site
B
dnaJ
B. subtilis
M. caseolyticus
S. auricularis
S. muscae
S. rostri
S. microti
S. chromogenes
S. hyicus
S. intermedius
S. delphini
S. pseudintermedius
S. lutrae
S. schleiferi schleiferi
S. schleiferi coagulans
S. felis
S. capitis capitis
S. capitis urealyticus
S. caprae
S. saccharolyticus
S. epidermidis
S. warneri
S. pasteuri
S. aureus anaerobius
S. aureus aureus
S. simiae
S. haemolyticus
S. hominis hominis
S. hominis novobiosepticus
S. lugdunensis
S. devriesei
S. cohnii cohnii
S. cohnii urealyticus
S. nepalensis
S. xylosus
S. succinus succinus
S. succinus casei
S. equorum equorum
S. equorum linens
S. saprophyticus bovis
S. saprophyticus saprophyticus
S. gallinarum
S. arlettae
S. kloosii
S. pettenkoferi
S. massiliensis
S. simulans
S. condimenti
S. carnosus carnosus
S. carnosus utilis
S. piscifermentans
S. lentus
S. pulvereri
S. vitulinus
S. fleurettii
S. sciuri sciuri
S. sciuri carnaticus
S. sciuri rodentium
S. stepanovicii
0.1 subs./site
C
rpoB
M. caseolyticus
S. muscae
S. microti
S. intermedius
S. delphini
S. pseudintermedius
S. rostri
S. felis
S. lutrae
S. chromogenes
S. hyicus
S. agnetis
S. schleiferi schleiferi
S. schleiferi coagulans
S. simulans
S. condimenti
S. carnosus carnosus
S. carnosus utilis
S. piscifermentans
S. hominis hominis
S. hominis novobiosepticus
S. lugdunensis
S. pettenkoferi
S. warneri
S. pasteuri
S. capitis capitis
S. capitis urealyticus
S. saccharolyticus
S. epidermidis
S. caprae
S. haemolyticus
S. devriesei
S. aureus anaerobius
S. simiae
S. cohnii cohnii
S. cohnii urealyticus
S. nepalensis
S. gallinarum
S. succinus succinus
S. succinus casei
S. equorum equorum
S. equorum linens
S. saprophyticus bovis
S. saprophyticus saprophyticus
S. xylosus
S. kloosii
S. arlettae
S. auricularis
S. sciuri sciuri
S. sciuri carnaticus
S. sciuri rodentium
S. lentus
S. stepanovicii
S. pulvereri
S. vitulinus
S. fleurettii
B. subtilis
0.1 subs./site
D
tuf
B. subtilis
S. aureus aureus
M. caseolyticus
S. auricularis
S. pettenkoferi
S. massiliensis
S. cohnii cohnii
S. cohnii urealyticus
S. nepalensis
S. gallinarum
S. saprophyticus bovis
S. saprophyticus saprophyticus
S. succinus succinus
S. succinus casei
S. xylosus
S. arlettae
S. equorum equorum
S. equorum linens
S. kloosii
S. simulans
S. condimenti
S. carnosus carnosus
S. carnosus utilis
S. piscifermentans
S. epidermidis
S. saccharolyticus
S. capitis capitis
S. capitis urealyticus
S. caprae
S. aureus anaerobius
S. simiae
S. haemolyticus
S. hominis hominis
S. hominis novobiosepticus
S. lugdunensis
S. devriesei
S. warneri
S. pasteuri
S. muscae
S. chromogenes
S. felis
S. intermedius
S. delphini
S. pseudintermedius
S. lutrae
S. schleiferi schleiferi
S. schleiferi coagulans
S. hyicus
S. agnetis
S. lentus
S. vitulinus
S. fleurettii
S. sciuri sciuri
S. sciuri carnaticus
S. sciuri rodentium
0.06 subs./site

Supplementary Figure S1. Gene trees for individual loci assessed in this study. Shown are Bayesian 50% majority rule phylograms for A) the 16S rRNA, B) *dnaJ,* C) *rpoB,* and D) *tuf* gene fragments. MrBayes was run under the same conditions as those used for concatenated analyses with evolutionary model specified for whole gene fragments in Supplementary Table S2. Numbers represent posterior probabilities with grey-filled circles representing a posterior support of 1.00.
