# Supplementary material for: Phylogenetic relationships among Staphylococcus species and refinement of cluster groups based on multilocus data
Source: BMC Evol Biol. 2012 Sep 6;12:171. doi: 10.1186/1471-2148-12-171 (PMC3464590; doi:10.1186/1471-2148-12-171)
Supplement: Additional file 4: Figure S2 — Bayesian inferences of phylogeny are highly reproducible, regardless of model employed. Shown are plots of post-burnin generational log likelihoods (lnL) from five representative partitioning strategies across triplicate concatenated BI runs (A); and duplicate BEST runs (B). All runs were highly reproducible regardless of methodology and partitioning strategy. [file 1471-2148-12-171-S4.pdf]

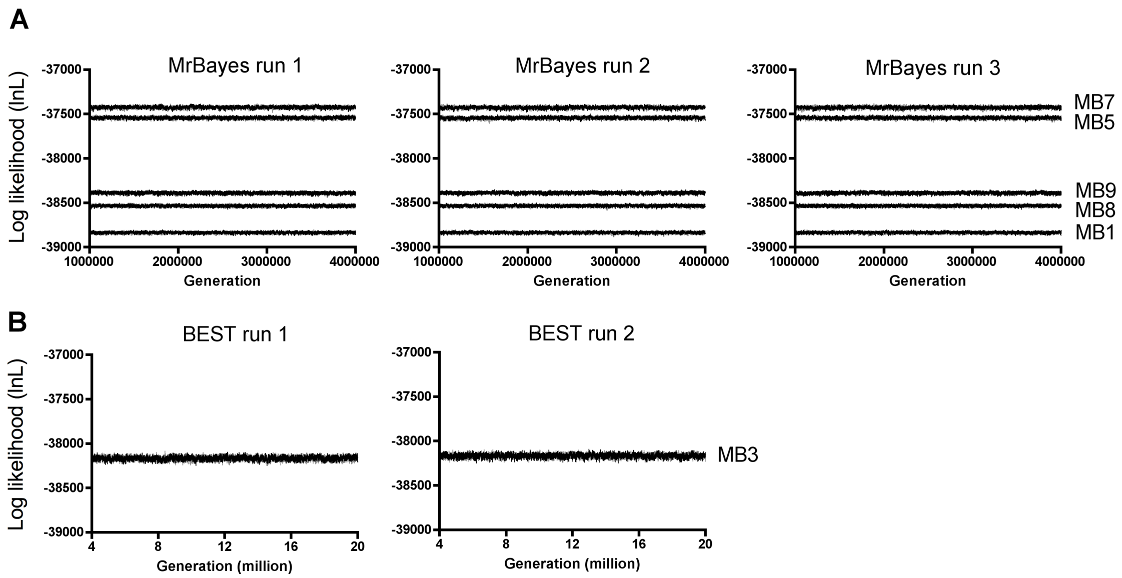

Supplementary Figure S2. Bayesian inferences of phylogeny are highly reproducible, regardless of model employed. Shown are plots of post-burnin generational log likelihoods (lnL) from five representative partitioning strategies across triplicate concatenated BI runs (A); and duplicate BEST runs (B). All runs were highly reproducible regardless of methodology and partitioning strategy.
