# Supplementary material for: Phylogenetic relationships among Staphylococcus species and refinement of cluster groups based on multilocus data
Source: BMC Evol Biol. 2012 Sep 6;12:171. doi: 10.1186/1471-2148-12-171 (PMC3464590; doi:10.1186/1471-2148-12-171)
Supplement: Additional file 5: Figure S3 — Tree length (TL) analysis indicates that overparameterization may be occurring within more highly partitioned datasets. Shown are post-burnin generational TL estimates for partitioning strategies assessed in this study. Note that as the complexity of partitioning increases evidence of increased TL and failed convergence is observed. [file 1471-2148-12-171-S5.pdf]

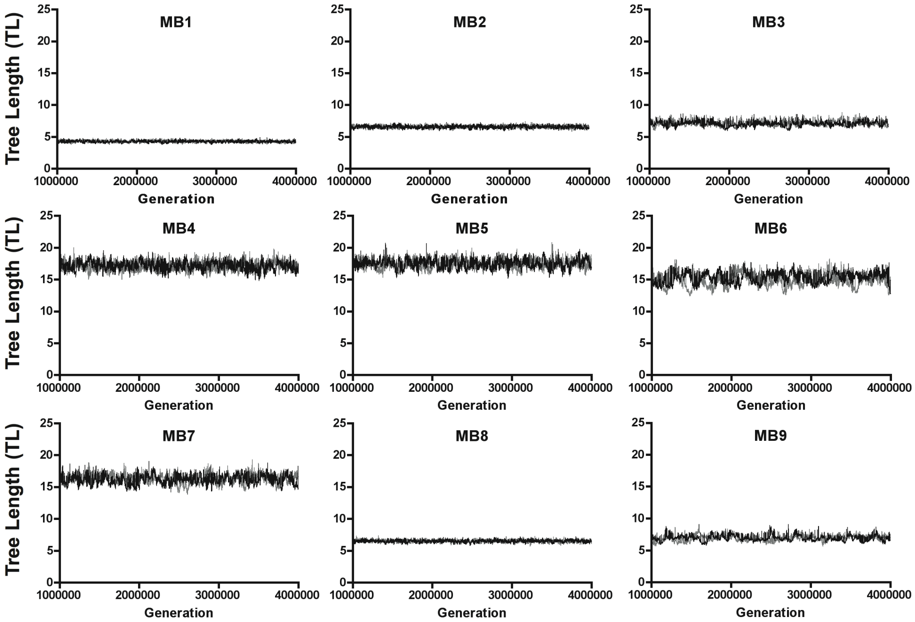

Supplementary Figure S3. Tree length (TL) analysis indicates that overparameterization may be occurring within more highly partitioned datasets. Shown are post-burnin generational TL estimates for partitioning strategies assessed in this study. Note that as the complexity of partitioning increases evidence of increased TL and failed convergence is observed.
