# Supplementary material for: Phylogenetic relationships among Staphylococcus species and refinement of cluster groups based on multilocus data
Source: BMC Evol Biol. 2012 Sep 6;12:171. doi: 10.1186/1471-2148-12-171 (PMC3464590; doi:10.1186/1471-2148-12-171)
Supplement: Additional file 6: Figure S4 — Model partitioning increases the mean tree length (TL) and run variance. Shown is a box plot indicating the mean TL and 95% confidence interval among partitioning strategies. [file 1471-2148-12-171-S6.pdf]

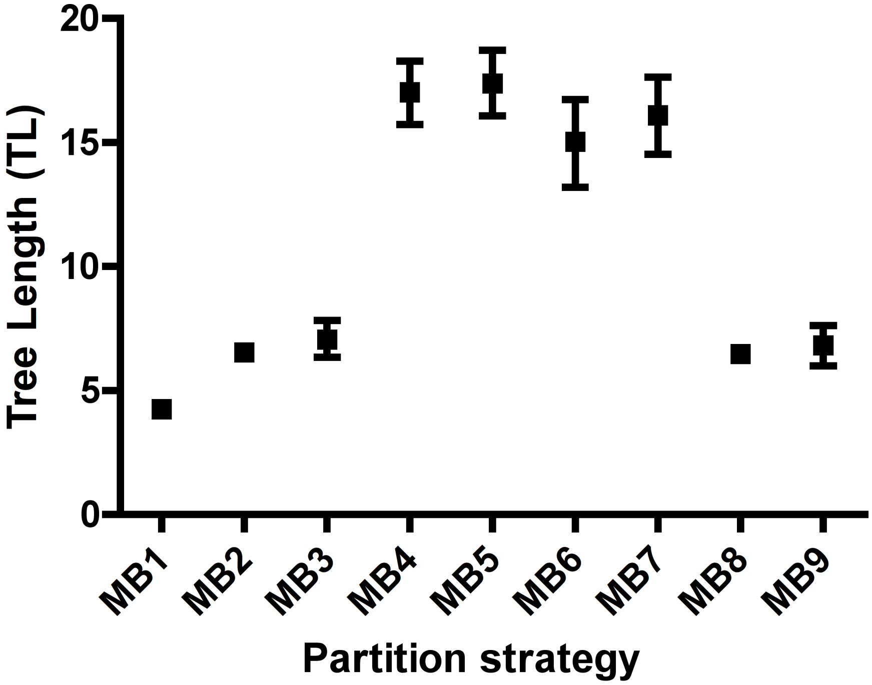

Supplementary Figure S4. Model partitioning increases the mean tree length (TL) and run variance. Shown is a box plot indicating the mean TL and 95% confidence interval among partitioning strategies.
