# Supplementary material for: Phylogenetic relationships among Staphylococcus species and refinement of cluster groups based on multilocus data
Source: BMC Evol Biol. 2012 Sep 6;12:171. doi: 10.1186/1471-2148-12-171 (PMC3464590; doi:10.1186/1471-2148-12-171)
Supplement: Additional file 7: Figure S5 — Inference of phylogeny using Bayesian estimation of species trees (BEST). Shown is a consensus phylogram of the staphylococcal species tree generated using all four gene fragments under the BEST methodology. Each gene fragment was treated as an individual locus for which individual gene trees were estimated (similar to MB3). Numbers represent posterior probabilities with grey-filled circles representing a posterior probability of 1.00. [file 1471-2148-12-171-S7.pdf]

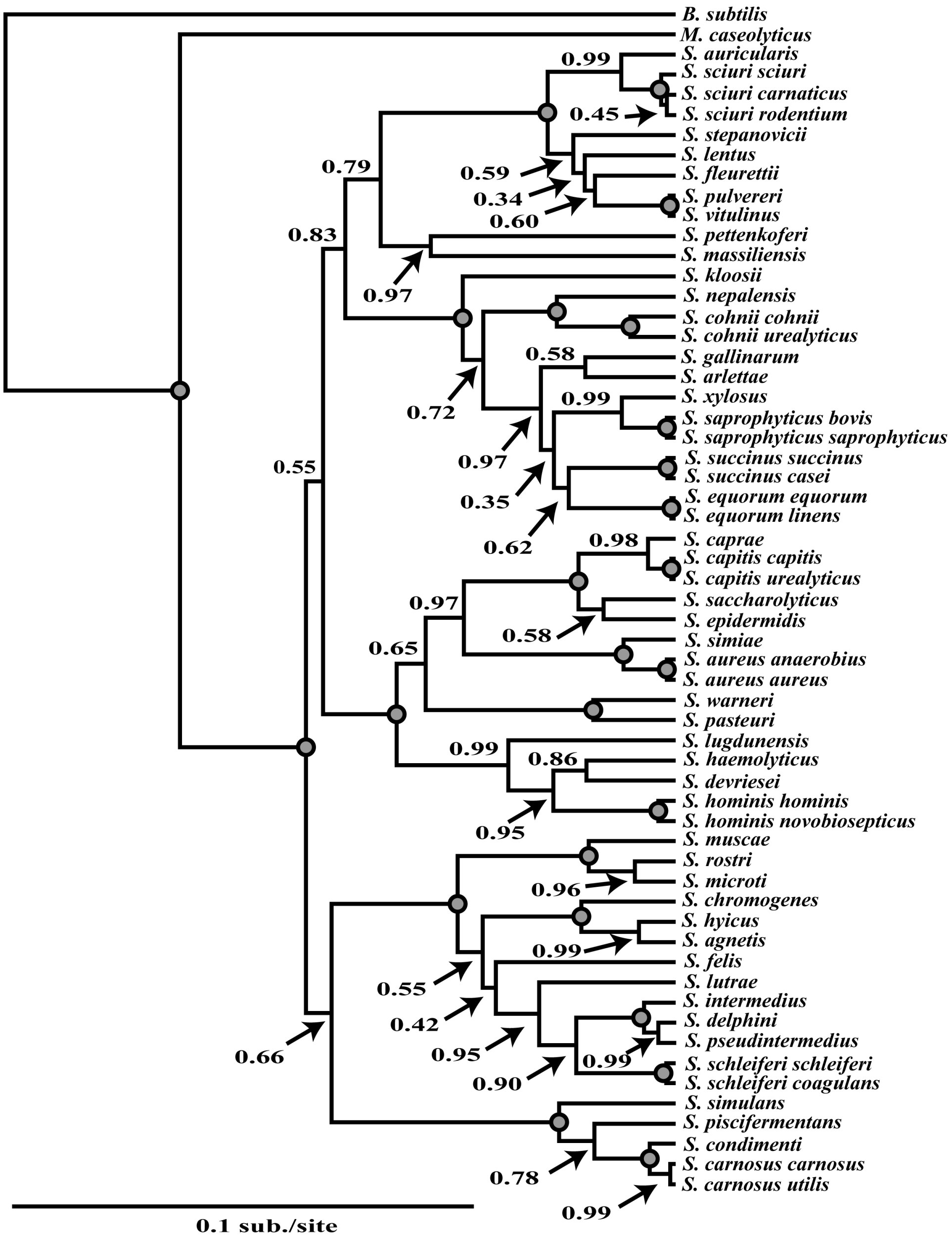

Supplementary Figure S5. Inference of phylogeny using Bayesian estimation of species trees (BEST). Shown is a consensus phylogram of the staphyloccal species tree generated using all four gene fragments under the BEST methodology. Each gene fragment was treated as an individual locus for which individual gene trees were estimated (similar to MB3). Numbers represent posterior probabilities with grey-filled circles representing a posterior probability of 1.00.
